# Insights Into Hepatic Sarcoidosis: Analysis of Histological Patterns, Hepatic Complications and Therapeutic Approaches

**DOI:** 10.1111/liv.70037

**Published:** 2025-02-19

**Authors:** Ludwig J. Horst, Katharina Zimmermann, Johanna Lutz, Sören Weidemann, Stefan Lüth, Ansgar W. Lohse, Julian Schulze zur Wiesch, Christoph Schramm, Malte H. Wehmeyer, Martina Müller, Marcial Sebode

**Affiliations:** ^1^ I. Department of Medicine University Medical Center Hamburg‐Eppendorf Hamburg Germany; ^2^ European Reference Network on Hepatological Diseases (ERN RARE‐LIVER) Germany; ^3^ Department of Internal Medicine I University Hospital Regensburg Regensburg Germany; ^4^ Institute for Pathology, University Medical Center Hamburg‐Eppendorf Hamburg Germany; ^5^ Department of Gastroenterology, Hepatology and Diabetology, Center of Internal Medicine II University Hospital Brandenburg an der Havel Brandenburg an der Havel Germany; ^6^ Martin Zeitz Center for Rare Diseases University Medical Center Hamburg‐Eppendorf Hamburg Germany

**Keywords:** cirrhosis, elevated liver enzymes, granulomatous liver disease, mini‐laparoscopy, portal hypertension

## Abstract

**Background and Aims:**

Sarcoidosis is a granulomatous multi‐systemic disorder of uncertain aetiology frequently involving the liver. This study aimed to delineate the histological characteristics, treatment effectiveness and factors predictive of liver‐related complications in individuals with hepatic sarcoidosis.

**Methods:**

This retrospective cohort study included patients diagnosed with hepatic sarcoidosis by liver biopsy, which was conducted at two tertiary care centres from January 2009 to December 2023. We analysed demographic, clinical and laboratory parameters, treatment response and outcome.

**Results:**

We enrolled 70 hepatic sarcoidosis patients with a median follow‐up of 45 months (IQR 11–97 months), including 37 males with a median age of 48 years (IQR 37–59 years). Elevated GGT (94%) and ALP (81%) were the most common liver‐specific biochemical alterations observed. Using mini‐laparoscopy for liver biopsy made it possible to macroscopically identify granulomatous disease in 71% of patients. While at baseline, 16% of the cohort showed evidence of potential portal hypertension, at the last follow‐up, 23% of patients developed complications related to portal hypertension. In addition to granulomatous changes, bile duct irregularities were found in 57% of liver biopsies, indicating cholangiopathy being part of the hepatic manifestation of sarcoidosis. Treatment with Ursodeoxycholic acid and prednisolone resulted in a significantly more pronounced decrease in ALT and ALP compared to untreated patients.

**Conclusions:**

Patients with hepatic sarcoidosis require careful assessment of disease manifestation with a particular focus on portal hypertension. Treatment with UDCA and prednisolone leads to a reduction of biochemical parameters in a significant proportion of these patients.

**Summary**

- The liver is an important site of manifestation of sarcoidosis and frequently presents with granulomas and alterations of bile ducts.
- However, little is known about the pathogenesis, clinical course and optimal management of these patients.
- Our results show that the disease course can be mild in some patients, while others develop complications such as portal hypertension with splenomegaly, ascites and oesophageal varices.
- Up to one‐third of our patients ultimately showed complications potentially related to portal hypertension.
- Ursodeoxycholic acid as an anti‐cholestatic drug and prednisolone as an immunosuppressive treatment can improve laboratory liver values in a considerable proportion of patients.
- It is unclear whether this laboratory improvement is associated with a better outcome.
- This remains to be shown in larger prospective studies and registries.

**Abbreviations**
18F‐FDG PET/CT: fluorine‐18‐fluorodeoxyglucose PET/computed tomography
ACE: angiotensin‐converting enzyme
AIH: autoimmune hepatitis
ALP: alkaline phosphatase
ALT: alanine aminotransferase
AST: aspartate aminotransferase
ATS: American Thoracic Society
AZA: azathioprine
BMI: body mass index
CCA: cholangiocarcinoma
CEUS: contrast‐enhanced ultrasound
CT: computed tomography
DILI: drug‐induced liver injury
DISR: drug‐induced sarcoid‐like reaction
FIB‐4: fibrosis‐4
GGT: gamma‐glutamyl transferase
HCC: hepatocellular carcinoma
IQR: interquartile range
METAVIR: meta‐analysis of histological data in viral hepatitis
MRI/MRCP: magnetic resonance imaging/Magnetic resonance cholangiopancreatography
MTX: methotrexate
NAS: non‐alcoholic fatty liver disease activity score
PBC: primary biliary cholangitis
PSC: primary sclerosing cholangitis
ROC: receiver operating characteristic
SLD: steatotic liver disease
SSC: secondary sclerosing cholangitis
TE: transient elastography
TIPS: transjugular intrahepatic portosystemic shunt
UDCA: Ursodeoxycholic acid
ULN: upper limit of normal

## 1 Introduction

Sarcoidosis is a multi‐systemic disease of unknown origin with frequent liver involvement and widespread appearance of granulomas derived from the reticuloendothelial system [1, 2, 3]. Current evidence suggests that the development of sarcoidosis is linked to genetic predisposition combined with environmental or occupational exposure to unknown substances, microbial antigens or medication [4, 5]. Hepatic manifestation of sarcoidosis can lead to chronic cholestasis and progressive fibrosis, but also to non‐cirrhotic portal hypertension [2, 6, 7, 8]. However, hepatic involvement is considered to be mild in the majority of affected patients [9]. In patients with hepatic sarcoidosis, detailed data on natural history, histological presentation, predictors of liver‐related morbidity and treatment are limited.

Serum levels of alkaline phosphatase (ALP) are regarded reliable biomarkers to assess liver involvement of sarcoidosis, and some authors suggest that the degree of liver‐test abnormalities is associated with advanced disease [10]. However, the diagnostic utility of non‐invasive markers for liver fibrosis, such as transient elastography (TE), the aspartate aminotransferase (AST)/alanine aminotransferase (ALT) ratio, or the fibrosis‐4 (FIB‐4) index, still needs to be analysed and validated for assessing the stage of hepatic sarcoidosis.

Steroids are considered first‐line treatment for hepatic sarcoidosis and improve clinical symptoms and liver function tests, but not necessarily histopathological findings [11, 12, 13]. Alternative long‐term treatment approaches encompass methotrexate (MTX) or azathioprine (AZA) [2, 6, 12, 13]. Furthermore, we and others have reported the efficacy of infliximab [14, 15]. Ursodeoxycholic acid (UDCA) as an anti‐cholestatic drug can also ameliorate increased liver enzymes and pruritus in hepatic sarcoidosis [2, 16]. For the subset of patients progressing to end‐stage liver disease, liver transplantation might become necessary [17]. Nonetheless, a previous study demonstrated significantly worse graft and patient survival rates following liver transplantation in patients with hepatic sarcoidosis compared to those with primary biliary cholangitis (PBC) or primary sclerosing cholangitis (PSC) [17].

Our study aimed to investigate the natural history of liver involvement, identify predictors of hepatic impairment, and determine the efficacy of different treatment regimens in a well‐defined cohort of patients with histologically confirmed hepatic sarcoidosis.

## 2 Patients and Methods

In this retrospective cohort study, data of consecutive patients who underwent ultrasound‐, surgically‐ or mini‐laparoscopically‐guided liver biopsy between January 2009 and December 2023 at one of the participating centers (University Medical Center Hamburg‐Eppendorf, Hamburg, Germany or University Hospital Regensburg, Regensburg, Germany) were systematically screened for the diagnosis of hepatic sarcoidosis.

Patients with a histologically‐confirmed diagnosis of hepatic sarcoidosis (presence of non‐caseating granulomas on histopathology), a compatible clinical presentation and exclusion of other granulomatous liver diseases such as tuberculosis, fungal infection, or PBC, were included in the analysis for this study according to the American Thoracic Society (ATS) guidelines [18]. In case of clinical or laboratory indications, patients were screened for viral hepatitis B or C infection, autoimmune hepatitis (AIH), drug‐induced liver injury (DILI), alpha‐1‐antitrypsin deficiency, hemochromatosis and Wilson's disease. Patients diagnosed with hepatic sarcoidosis were evaluated for extrahepatic manifestations.

Patients' demographics, encompassing environmental and occupational exposures, the diagnostic pathways leading to hepatological evaluation, as well as clinical, radiological, and laboratory data were collected retrospectively from the time of liver biopsy and throughout the follow‐up period until December 2023. The grade of liver fibrosis was assessed according to the meta‐analysis of histological data in viral hepatitis (METAVIR) score. Portal hypertension was suspected in patients with a combination of splenomegaly (> 120 mm) with signs of either oesophageal varices, portal hypertensive gastropathy, ascites or thrombocytopenia.

The study was approved by the Ethics Committee of the Hamburg Medical Association (2024‐101318‐BO‐ff). The collaborating center, the University Hospital Regensburg, received approval from the Institutional Review Board of the University Hospital Regensburg (24‐3692‐104). The study was performed in accordance with the ethical principles of the Declaration of Helsinki.

### 2.1 Statistical Analysis of Patients' Baseline Parameters and Follow‐Up Data

Statistical analyses were performed using SPSS (version 28.0.1.0; IBM, Armonk, NY, USA), GraphPad Prism (version 10; GraphPad Software Inc., La Jolla, CA, USA) and R (4.3.2, R Foundation for Statistical Computing, Vienna, Austria). The median and interquartile range (IQR; Q_1–_Q_3_) were used to describe continuous variables, while frequencies and percentages (%) were used to characterise categorical variables. Differences between two independent groups were assessed using the Mann–Whitney *U* test. Comparisons involving two dependent groups with quantitative variables were conducted through the Wilcoxon signed‐rank test. Spearman's rank correlation coefficient was used to perform univariate correlation analysis. A *p* < 0.05 was considered statistically significant.

## 3 Results

### 3.1 Clinical Features and Comprehensive Diagnostic Evaluation of Patients With Hepatic Sarcoidosis

We identified 70 patients in whom the diagnosis of hepatic sarcoidosis was confirmed by liver biopsy. The main baseline characteristics of the patients enrolled in this study are listed in Table 1. Hepatological work‐up was most frequently initiated by general practitioners or specialists in internal medicine (40%), followed by referring hospitals (9%), pulmonologists (4%), gastroenterologists (4%), emergency departments (4%), surgical departments (3%), and gynaecologists (1%). Patients were primarily evaluated for elevated liver enzymes and unknown sarcoidosis (67%) or suspected hepatic involvement of known extrahepatic sarcoidosis (28%; Figure S1).

**TABLE 1:** Characteristics of patients with hepatic sarcoidosis (AST/ALT: ULN 50 U/L (♂), 35 U/L (♀); GGT: ULN 65 U/L (♂), 38 U/L (♀); ALP: ULN 129 U/L (♂), 104 U/L (♀)).

Characteristics		Available data (*n*)
Age at baseline (years), median (IQR)	48 (37–59)	70/70
Male sex, *n* (%)	37 (53)	70/70
BMI (kg/m^2^), median (IQR)	27 (24–31)	64/70
Isolated hepatic sarcoidosis, *n* (%)	14 (20)	
Extrahepatic involvement, *n* (%)		
Pulmonary	47 (67)	70/70
Lymphatic	29 (43)	
Splenic	21 (31)	
Peritoneal	8 (12)	
Gastrointestinal	7 (11)	
Other (ocular, renal, heart, parotid, thyroidal, joints, nasal)	15 (21)	
Staging of pulmonary involvement, *n* (%)		
0	9 (20)	37/47
I	13 (28)	
II	17 (37)	
III	6 (13)	
IV	1 (2)	
Clinical presentation, *n* (%)		
Asymptomatic	21 (32)	65/70
Fatigue	21 (32)	
Weight loss	16 (25)	
Abdominal pain	15 (23)	
Night sweats	11 (17)	
Pruritus	2 (3)	
Fever	9 (14)	
Baseline diagnostics		
Laboratory parameters (U/L), median (IQR)		
AST	41 (27–58)	68/70
ALT	52 (31–81)	67/70
GGT	207 (133–416)	63/70
ALP	186 (121–284)	68/70
ACE	66 (31–113)	59/70
sIL–2R (U/mL)	845 (488–1307)	29/70
Patients with laboratory parameters > ULN, *n* (%)		
AST	31 (46)	
ALT	42 (63)	
GGT	59 (94)	
ALP	55 (81)	
Imaging, *n* (%)		
Splenomegaly	38 (64)	59/70
Coarsening and inhomogeneity of the liver parenchyma	40 (57)	70/70
Hepatomegaly	17 (25)	68/70
Hypodense nodular lesions in the liver	18 (27)	67/70
No detectable abnormalities	12 (17)	70/70
Transient elastography (kPa), median (IQR)	7.3 (5.7–10.6)	37/70
Length of follow‐up (months), median (IQR)	45 (11–97)	70/70

In total, 37 patients were male, and the median age was 48 years. Hepatic comorbidities were found in 18 patients. Ten patients with available information exhibited histological signs indicative of steatotic liver disease (SLD), with mildly elevated non‐alcoholic fatty liver disease activity scores (NAS). Among these patients, eight had a NAS of 1, one had a NAS of 2, and one had a NAS of 4. In two patients, a history of alcohol misuse was reported. However, histological signs in this regard were absent. Three patients had chronic viral hepatitis B infection (two patients with undetectable viral load under antiviral treatment, one patient with low replication rate) and two patients had a history of viral hepatitis C infection. One patient was diagnosed with DILI due to phenprocoumon 8 years before the diagnosis of sarcoidosis.

73% of patients took one or more medications at the time of inclusion. The most common medication was insulin (13%), followed by acetylsalicylic acid and pantoprazole (each 11.4%). A detailed list of concomitant medication is provided in Table S4. No increased prevalence for specific allergies, intolerances, occupational exposures, or illegal drug use was detected among the included patients.

At baseline, all patients exhibited either biochemical irregularities, imaging results consistent with sarcoidosis, or an elevated liver stiffness determined by TE (FibroScan), which led to the indication to perform liver biopsy. Laboratory assessment showed abnormalities of liver enzymes in all participants with available data at baseline. Most patients displayed a mild to moderate increase in cholestatic liver enzymes (Table 1). Elevated GGT levels were observed in 94% of patients, while 81% had ALP levels exceeding the upper limit of normal (ULN). At baseline, 29% of the cohort presented with a solely cholestatic liver enzyme pattern with normal levels of AST and ALT. Angiotensin‐converting enzyme (ACE) levels were elevated in 50% of cases. The most frequent direct and indirect findings on imaging (ultrasound or magnetic resonance imaging) supporting liver disease were unspecific parenchymal abnormalities of the liver (coarsening/inhomogeneity) and splenomegaly. Only 27% of patients had signs of hypodense nodular lesions on imaging (Table 1).

TE at baseline showed a median liver stiffness of 7.3 kPa (IQR 5.7–10.6 kPa). The examined cohort exhibited the following distribution of histological fibrosis grades: F0 in 54% (35/65), F1 in 26% (17/65), F2 in 8% (5/65), F3 in 5% (3/65), and liver cirrhosis in 8% (5/65) of patients.

At baseline, male patients, despite comparable age and body mass index (BMI), tended to have significantly higher levels of AST, ALT and GGT than female patients with sarcoidosis (Table S5). Furthermore, male patients had higher liver stiffness measurements (9.6 kPa, IQR 7.03–14.65 kPa) compared to female patients (5.8 kPa, IQR 5.09–7.34 kPa; *p* = 0.002). Additionally, males with hepatic sarcoidosis exhibited a higher prevalence of splenomegaly (78% vs. 48%) and were more frequently asymptomatic (40% vs. 23%) than women.

The median duration of follow‐up from histological detection of hepatic sarcoidosis to the last follow‐up was 45 months (IQR 11–97 months). An overview of the proportion of patients with available baseline and follow‐up data from the overall cohort can be found in Table S1.

Four patients developed extrahepatic neoplasia (Hodgkin lymphoma, B‐cell lymphoma, breast cancer and kidney cancer) during the observation period (median age 60.3 years [IQR 51.2–69.0 years]). Budd–Chiari syndrome was diagnosed in another patient during the course of the disease with confirmation of a methylenetetrahydrofolate reductase mutation. Serious cardiovascular events occurred in seven patients at a median age of 57.5 years (IQR 44.8–64.3 years); six of them showed signs of coronary heart disease, and one developed myocardial infarction. One patient died during follow‐up in the context of acute respiratory decompensation of pulmonary hypertension. Liver values were normal in this patient.

### 3.2 Mini‐Laparoscopy as an Additional Diagnostic Tool in Patients With Suspected Granulomatous Liver Disease

**FIGURE 1:**
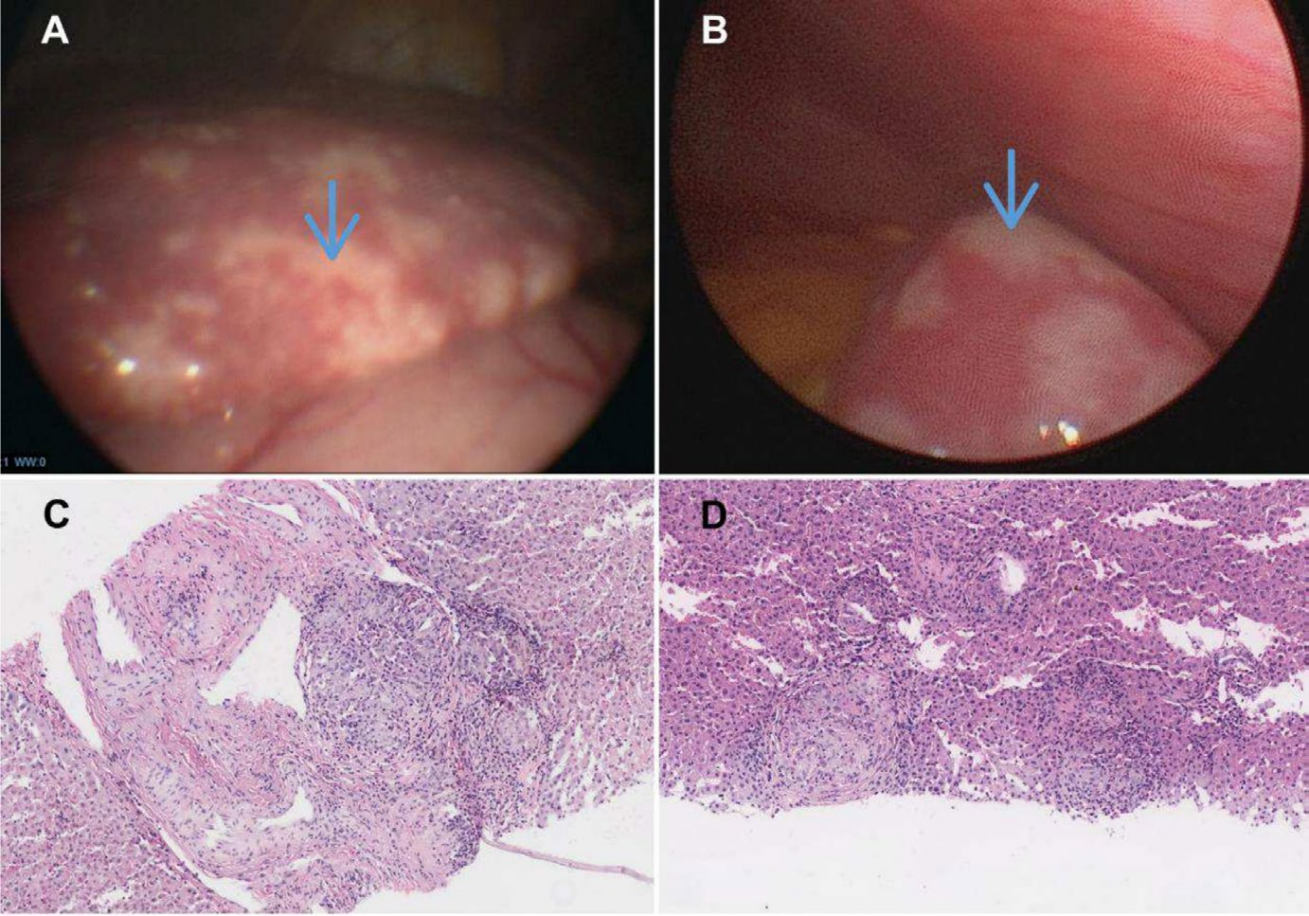
Visualisation of the liver (A) and spleen (B) during mini‐laparoscopy with multiple granulomatous lesions on the surfaces of the organs (blue arrows). Liver histology revealed portal (C) and lobular (D) epithelioid cell granulomas (haematoxylin–eosin staining, ×200).

In 37 of 70 (53%) patients, liver biopsies were obtained during mini‐laparoscopy, and in three patients (4%), samples were obtained during surgical interventions (see supplementary patients and methods). In the group of patients with a visual assessment of the abdominal cavity, 71% had macroscopic evidence of granulomatous liver disease (multiple whitish lesions, Figure 1). Additionally, mini‐laparoscopy revealed macroscopic signs of splenic and peritoneal granulomas in 17% and 14% of the cohort, respectively. In 15 of 37 (41%) patients who underwent liver biopsy by mini‐laparoscopy, biopsies of both liver lobes were obtained. Among these, 27% demonstrated granulomas on histology in only one of the two biopsies.

### 3.3 Beyond Granulomas: Cholangiopathic Changes in Hepatic Sarcoidosis

Predominant histological findings included epithelioid, non‐caseating granulomas in 86% and giant cell granulomas in 51% of patients. In most cases, the granulomas were multifocally distributed with lobular involvement in 70%, localisation in the portal or periportal area in 60% and/or biliary duct association in 40% of cases. The location of granulomas was not associated with the degree of AST or ALP elevation or with liver stiffness assessed by TE (Table S2).

In addition to the granulomatous changes, 57% of patients with hepatic sarcoidosis further revealed irregularities of the bile ducts. Among these, ductopenia was present in 15%, whereas 28% showed bile duct proliferation, and 37% of cases exhibited unspecified changes in bile ducts. A subgroup of 14% of patients showed advanced bile duct damages resembling sclerosing cholangitis in histology and/or magnetic resonance imaging/magnetic resonance cholangiopancreatography (MRI/MRCP). Histological irregularities in this group included advanced degenerative changes in the interlobar bile ducts with signs of biliary outflow obstruction and extensive ductular reaction. This was reflected by elevated GGT and ALP values in the aforementioned patients at baseline. None of these patients showed evidence of high‐grade fibrosis (> F2). Despite the changes, there was no evidence of acute decompensation or serious adverse events, such as cholangitis, in this subgroup during follow‐up.

### 3.4 Portal Hypertension as a Major Complication of Hepatic Sarcoidosis

At the time of inclusion, 64% of the cohort had moderate to severe splenomegaly with a median length of 128 mm (IQR 110–145 mm). Of these, 74% showed no or only moderate liver tissue damage (F0–F2). Further clinical or laboratory signs of portal hypertension, such as ascites, oesophageal varices, portal hypertensive gastropathy or thrombocytopenia, were found in 16% of the cohort with available data. Patients with signs of portal hypertension were characterised by significantly higher FIB‐4 scores (=Age (years) × AST (U/L)/[Platelets (10^9^/L) × ALT^1/2^ (U/L)]) at baseline, indicating its potential diagnostic value over individual laboratory values (Table 2, Figure S2).

**TABLE 2:** Comparison of clinical and laboratory characteristics between patients with and without liver cirrhosis or portal hypertension (n. a., not applicable).

	Cirrhosis			Portal hypertension		
	Yes	No	*p*	Yes	No	*p*
Age (years), median (IQR)	62 (48–63)	47 (36–58)	0.095	54 (40–62)	48 (36–57)	0.507
AST (U/L), median (IQR)	45 (27–103)	41 (28–58)	0.693	75 (41–112)	39 (27–52)	0.015
ALP (U/L), median (IQR)	170 (126–665)	191 (126–284)	0.765	312 (174–589)	181 (112–274)	0.048
AST/ALT ratio, median (IQR)	1.1 (0.8–1.6)	0.7 (0.6–1.1)	0.272	1.1 (0.6–1.9)	0.7 (0.6–1.1)	0.091
FIB‐4, median (IQR)	2 (1.4–5.4)	1.1 (0.7–1.7)	0.024	4.6 (1.9–7.1)	1 (0.7–1.4)	< 0.001
Albumin (g/L), median (IQR)	37 (34–45)	38 (34–42)	0.889	37 (31–44)	38 (34–42)	0.785
INR, median (IQR)	1 (1–1.1)	1 (1–1.1)	0.514	1.1 (1–1.2)	1 (1–1.1)	0.029
ACE (U/L), median (IQR)	n. a.	71 (33–115)	n. a.	99 (32–132)	58.7 (27–112)	0.402
sIL‐2R (U/mL), median (IQR)	n. a.	845 (519–1601)	n. a.	n. a.	817 (483–1294)	n. a.

Four patients developed signs of new‐onset portal hypertension during follow‐up. Thus, a total of 23% of the cohort with available data showed signs of portal hypertension at the last follow‐up. One patient presented with new‐onset ascites, one patient with first manifestation of portal hypertensive gastropathy and thrombocytopenia, and two patients with splenomegaly and thrombocytopenia. All patients with new‐onset portal hypertension received immunosuppressive therapy during the observation period (prednisolone in three patients, prednisolone in combination with azathioprine in another patient). Two patients showed a good response to therapy. For two patients, no laboratory data were available during this period. Three patients with known portal hypertension (fibrosis stages 2, 3 and 4) underwent implantation of a transjugular intrahepatic portosystemic shunt (TIPS). Two patients obtained the intervention in close proximity to the diagnosis, after 0–1 months, and one patient received a TIPS implantation after 50 months. Other critical adverse events related to complications of portal hypertension (e.g., variceal bleeding, spontaneous bacterial peritonitis) did not occur during follow‐up.

### 3.5 Treatment Response and Follow‐Up of Patients With Hepatic Sarcoidosis

In total, 74% of the cohort received treatment for sarcoidosis. In 62% of those patients, therapy was initiated directly after the diagnosis of hepatic sarcoidosis. 22% of patients had at least 6 months of follow‐up before initiation of therapy, and 26% of the total cohort never received therapy. 16% of patients received therapy for extrahepatic manifestations of sarcoidosis before the diagnosis of hepatic involvement and were not included in the analyses of the initial treatment response.

During the spontaneous course of hepatic sarcoidosis, in patients who never received treatment or had a secondary therapy induction, no significant increases in AST, ALT, GGT or ALP were detected. However, clinically relevant spontaneous biochemical improvement was not evident either (Figure 3, Table S3). None of these patients without therapy showed signs of acute hepatic decompensation.

**FIGURE 2:**
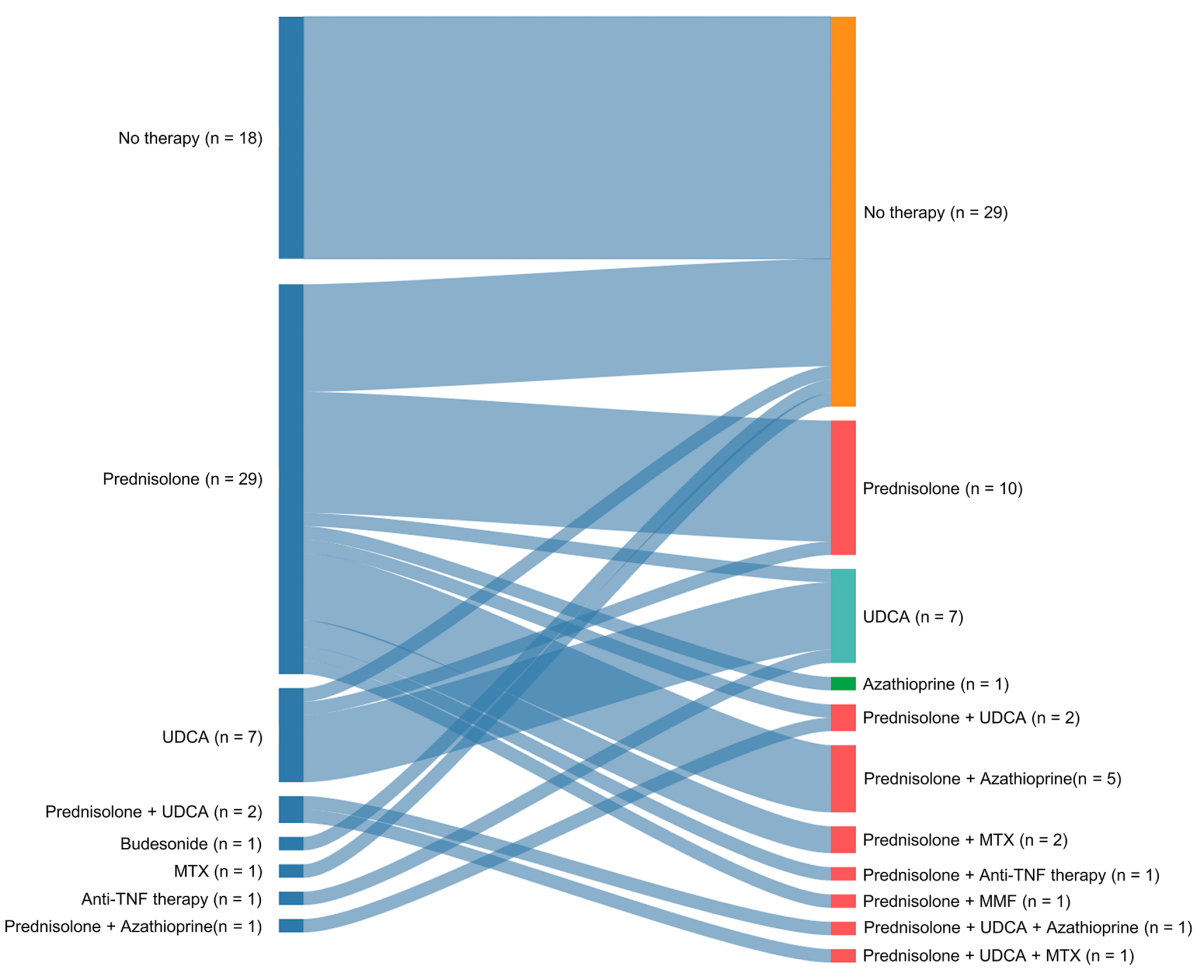
Illustration of initial and subsequent treatment regiments with changes in therapy.

Patients receiving therapy for hepatic sarcoidosis showed a wide range of therapeutic regimens. The various initial and subsequent treatment options are displayed in Figure 2.

**FIGURE 3:**
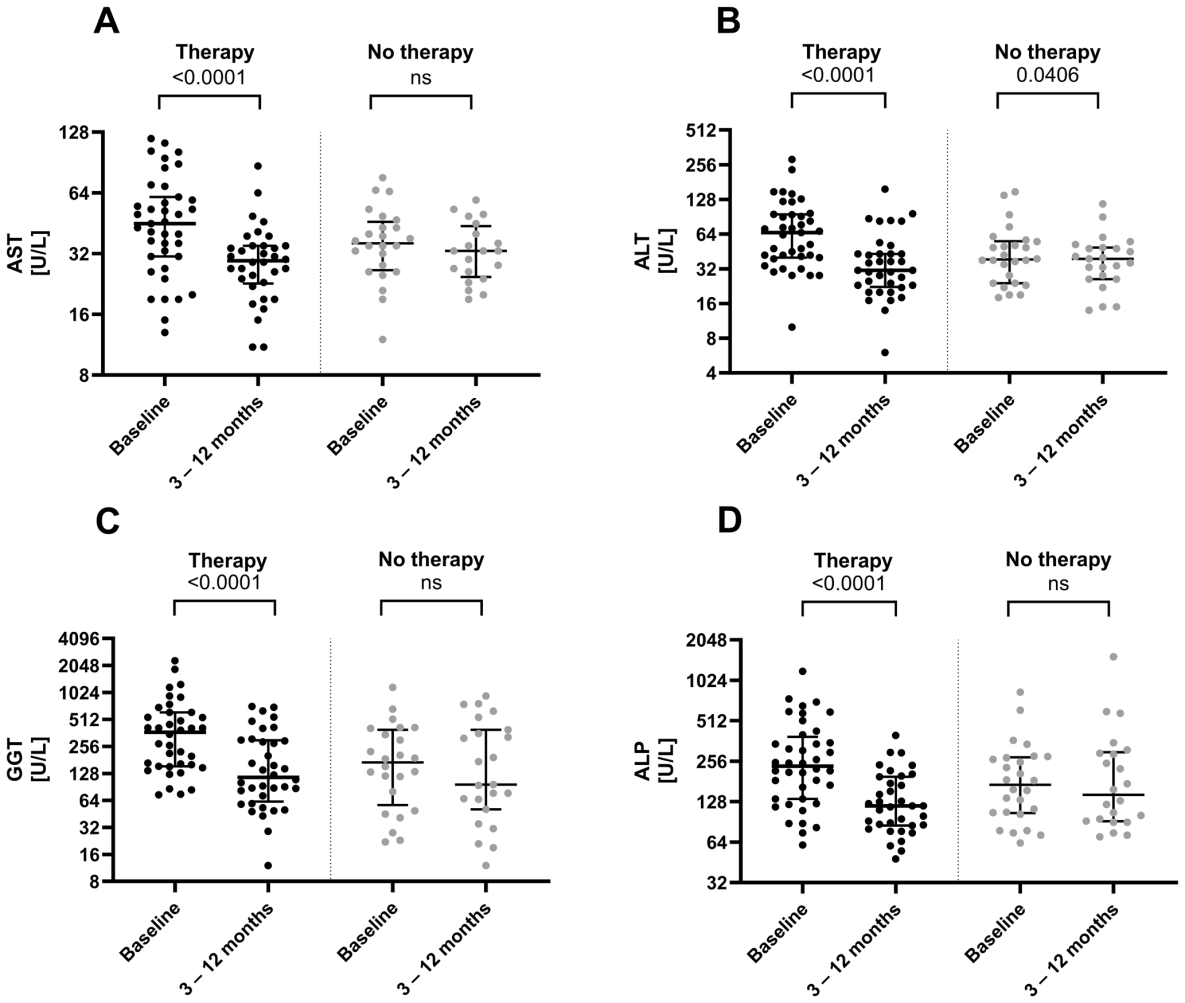
Difference in the median levels of AST (A), ALT (B), GGT (C) and ALP (D) of patients with hepatic sarcoidosis between baseline and 3–12 month follow‐up either with or without treatment (ns, not significant).

Patients receiving treatment for hepatic sarcoidosis (immunosuppression or anti‐cholestatic therapy) showed a marked biochemical response with significant declines in AST (*p* < 0.0001), ALT (*p* < 0.0001), GGT (*p* < 0.0001) and ALP (*p* < 0.0001) levels within the first 12 months compared to baseline (Figure 3). Furthermore, eight out of twelve patients with primary therapy initiation and repeated TE experienced a median decrease in liver stiffness of −1.5 kPa (IQR 4–0.8 kPa) after 12–24 months, although this did not reach significance (*p* = 0.136).

**FIGURE 4:**
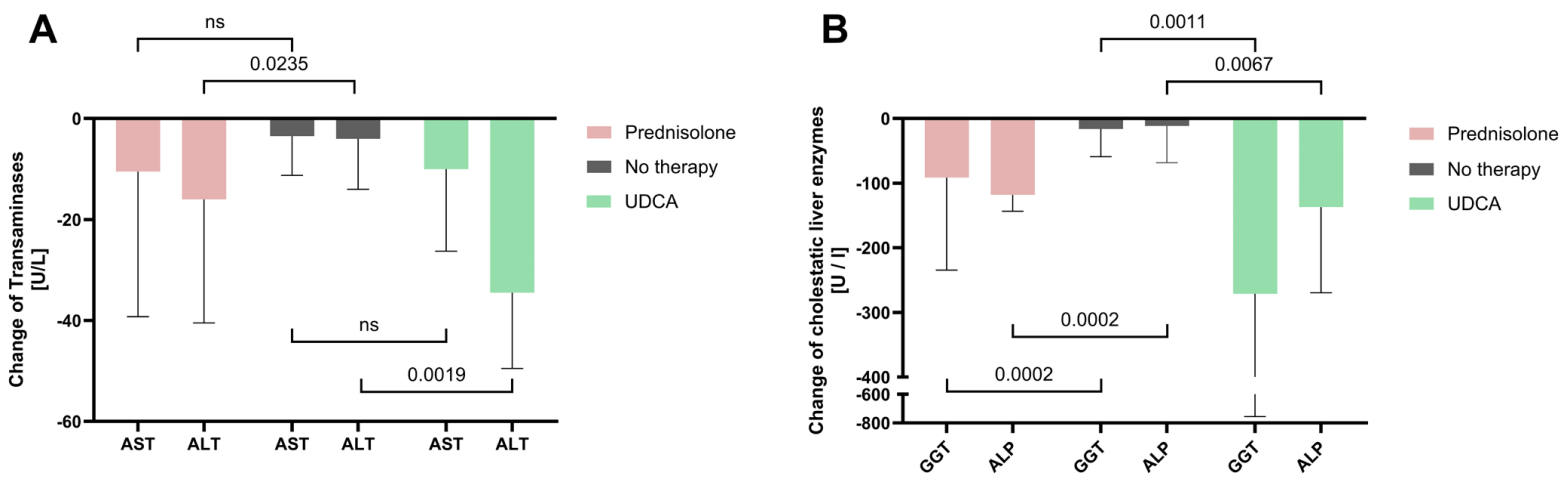
Comparison of the median changes between baseline and follow‐up after 3–12 months in AST/ALT (A) and GGT/ALP (B) in patients without therapy and established therapy (Prednisolone or UDCA; ns, not significant).

Prednisolone or UDCA monotherapy was the most common initial treatment in 29 and seven patients, respectively. There was no significant difference in the levels of transaminases and cholestasis parameters between the two groups at baseline. Comparing these treatments, both, patients under prednisolone or UDCA showed a reduction of liver parameters over time (Figure 4 and Table S3).

In patients initially treated with prednisolone monotherapy, therapy led to a significant reduction in GGT (*p* < 0.0001), ALP (*p* < 0.0001), AST (*p* = 0.0045) and ALT (*p* = 0.0011) after 3–12 months. Compared to the spontaneous course of the disease, patients on prednisolone therapy showed significantly larger median reductions in GGT (*p* = 0.0002) and ALP (*p* = 0.0002) within the first year of treatment (Figure 4). Although only a minority of patients showed normalisation of transaminases (AST: 41%, ALT: 33%) and cholestatic parameters (GGT: 17%, ALP: 29%) during the follow‐up period, a substantial proportion of patients experienced a decrease by more than 25% of baseline in AST (55%), ALT (71%), GGT (75%) and ALP (79%) within 12 months.

In patients with hepatic sarcoidosis, UDCA monotherapy led to a significant reduction in ALT (*p* = 0.03) and the cholestatic parameters GGT (p = 0.03) and ALP (p = 0.03) within the first year. Accordingly, the median reduction in ALT (*p* = 0.002), GGT (*p* = 0.001), and ALP (*p* = 0.007) was found to be significantly larger than in untreated patients (Figure 4, Table S3). Under UDCA treatment, a marked reduction in AST, ALT, GGT and ALP by more than 25% of the baseline value could be achieved in the majority of the patients (67%, 100%, 100% and 83%, respectively) within 12 months. Although normalisation of AST and ALT was achieved in 67% and 83% of those treated with UDCA monotherapy within 3–12 months, normalisation of GGT or ALP was observed in 0% and 33%, respectively.

## 4 Discussion

Here, we present data from a large cohort of patients with histologically confirmed hepatic sarcoidosis. We provide insights into diagnostic pathways and the clinical course and therapy for hepatic sarcoidosis, encompassing aspects of histopathology, assessment of portal hypertension, sex differences, the presence of concomitant cardiovascular comorbidities, and therapeutic approaches.

Hepatic sarcoidosis continues to represent a diagnostic challenge. In our study, 67% of patients were diagnosed following diagnostic work‐up for elevated liver enzymes in the absence of previously known sarcoidosis, underscoring the importance of including hepatic sarcoidosis in the differential diagnosis of elevated liver enzymes.

Given the complexity and the limited understanding of this disease, it appears advisable to assess potential external exposures and triggers, along with associated comorbidities, already during the initial evaluation phase. Although studies indicate that the route and type of environmental or occupational exposure may influence the specific organs affected by sarcoidosis, with liver involvement more prevalent in individuals with livestock contact or occupations that entail close human interaction, an association with external triggers could not be detected in the present investigation [4, 19, 20, 21]. However, this was not the specific aim of this study. Drug exposure has been linked to a systemic sarcoid‐like disease. These drug‐induced sarcoid‐like reactions (DISR) typically emerge after the initiation of the causative drug and often resolve upon its discontinuation [22]. Due to the similarities between sarcoidosis and DISR, it remains unclear whether DISR represents a ‘true’ manifestation of sarcoidosis or an “independent” granulomatous syndrome [22, 23]. In the current study, we could not conclusively identify concomitant drugs potentially associated with the development of hepatic sarcoidosis.

Previous studies revealed a higher incidence of cardiovascular diseases and an elevated risk of coronary artery disease in sarcoidosis patients [24, 25]. In our cohort, serious cardiovascular events were observed in seven patients; six exhibited signs of coronary heart disease, while one experienced a myocardial infarction. With a median age at onset of 57.5 years (IQR 44.8–64.3 years), cardiovascular events were observed at an age slightly lower than that typically reported in Western countries, where the onset of coronary artery disease occurs at a median age of 65 years [26]. The current findings and data on cardiovascular risk in hepatic sarcoidosis should be interpreted with caution, not only due to the small cohort size but also because this study did not systematically evaluate other cardiovascular risk factors, such as hypertension, smoking, and a family history of coronary heart disease.

Histological confirmation remains the gold standard for diagnosing hepatic sarcoidosis, yet there are no recommendations for specific liver biopsy techniques. This study highlights the potential advantages of mini‐laparoscopy for liver biopsy in cases of suspected granulomatous liver disease, surpassing the limitations of percutaneous liver biopsy in the Menghini technique. More than 2/3 of patients who underwent mini‐laparoscopy had macroscopic evidence of granulomatous liver disease, demonstrating the higher sensitivity of mini‐laparoscopy for detecting granulomatous disease compared to conventional imaging (ultrasound, MRI) with only 27% of our cohort showing evidence of hepatic granulomas. Furthermore, mini‐laparoscopy offers a reliable assessment of the different abdominal manifestations of sarcoidosis. During mini‐laparoscopy, liver biopsy was guided by the macroscopic detection of granulomas on the liver surface and was obtained from both liver lobes to minimise sampling error, if feasible [27]. In 27% of patients, histopathological differences in the presence of diagnosis‐confirming granulomas were observed between the left and right liver lobes. Past reports have noted the susceptibility to sampling errors with conventional single liver biopsy for granulomatous liver disease [28]. The additional diagnostic benefit of mini‐laparoscopy by reducing false negative diagnostic test results and assessing further sarcoidosis‐affected abdominal organs supports its potential role in the diagnostic work‐up of patients with suspected hepatic sarcoidosis.

In our cohort, only 27% of patients with hepatic sarcoidosis exhibited hypodense nodular lesions on imaging which is in line with a previous study by Graf et al. [2]. 17% of patients in our cohort and 14.5% in the Graf et al. study did not show any abnormalities [2]. Advanced imaging modalities, such as contrast‐enhanced ultrasound (CEUS) or Fluorine‐18‐fluorodeoxyglucose PET/computed tomography (18F‐FDG PET/CT) can enhance the detection rate of hepatic or splenic isoechoic nodules undetectable by conventional ultrasound and should be considered in eligible patients [29, 30, 31]. However, imaging techniques such as 18F‐FDG PET/CT currently lack disease‐specific characteristics and are often limited by their high costs and limited availability.

Laboratory evidence of cholestasis was observed in up to 94% of patients in this study, serving as a key indicator of liver involvement prior to histological confirmation. It is considered that cholestasis in sarcoidosis is primarily of intrahepatic origin and is caused by progressive compression of the bile ducts by portal and periportal granulomas [7, 32, 33]. Sedki et al. reported that non‐caseating granulomas are distributed in a portal or multifocal pattern [34]. Our study provides a more detailed analysis of the histological distribution of liver granulomas, with lobular involvement in 70%, localisation in the portal or periportal area in 60% and/or biliary duct association in 40% of cases. However, our study could not conclusively establish a direct correlation between granuloma location in the liver and the severity of cholestatic liver values. Conversely, almost 60% of patients exhibited bile duct changes such as ductopenia or bile duct proliferation, suggesting possible further, non‐granulomatous factors contributing to sarcoidosis‐induced bile duct damage.

At baseline, 64% of cases displayed clinical signs of portal hypertension with present splenomegaly. In the majority of these cases (74%) high‐grade fibrosis (> F2) was absent, supporting a non‐cirrhotic mechanism of portal hypertension. Associated complications, such as thrombocytopenia, ascites, oesophageal varices, and/or portal hypertensive gastropathy were found in 16% of the total cohort. While this result at baseline is lower compared to rates of up to 30% reported in previous studies, our data underscores a potential progression of hepatic sarcoidosis‐associated portal hypertension over time from 16% to 23% of our cohort at the last follow‐up [2, 34]. Thus, our study highlights that relevant disease progression can potentially manifest in a subset of patients. This increase substantiates the recommendation by Graf et al. [2] for regular screening for portal hypertension in patients with hepatic sarcoidosis. The lower rate of patients with portal hypertension in our study at baseline is most likely due to diagnosis at an earlier stage. This is also reflected by a lower proportion of patients with cirrhosis (8%) compared to other studies, in which cirrhosis was present in up to 25% of patients [2, 32, 34, 35, 36]. In contrast to previous studies, our data indicate that single laboratory parameters are less reliable in identifying patients with advanced sarcoid liver disease [10, 34]. However, a combination of laboratory parameters could potentially help to detect advanced disease stages. In our study, the FIB‐4 score correlated with the presence of complications of portal hypertension and might hence be useful to identify patients at risk for decompensation. Although Graf et al. [2] recommend cross‐sectional imaging and endoscopic evaluation to assess signs of portal hypertension in hepatic sarcoidosis patients, our data support the use of the FIB‐4 score as an additional non‐invasive tool to identify patients at increased risk. A FIB‐4 score > 1.95 indicated an increased risk for portal hypertension in our cohort.

The sex distribution in hepatic sarcoidosis remains a subject of debate, with studies inconsistently reporting a predominance of either males or females, or finding balanced ratios between the sexes [2, 6, 34, 37, 38, 39, 40, 41]. Our data indicate that men not only present with higher laboratory values indicative of increased liver inflammation (AST, ALT) and higher liver stiffness but also display more frequently signs of portal hypertension, such as splenomegaly, at the time of enrolment. These findings align with previous observations, that show that male patients more often present with advanced liver involvement [32, 42].

Current therapeutic options for hepatic sarcoidosis include either a watch‐and‐wait approach, treatment with immunosuppression or UDCA. Nonetheless, evidence‐based recommendations are missing, and assessing the need for treatment is considered difficult due to cases with spontaneous remission [43, 44]. However, since neither clinically relevant spontaneous biochemical improvement nor liver stiffness decrease was observed in our cohort, future studies need to focus more on therapeutic options and their impact on outcomes. Our study revealed a significant reduction in transaminases and cholestatic parameters in patients receiving either immunosuppressive and/or anti‐cholestatic therapy. Additionally, eight out of twelve patients with primary therapy and available TE showed a nonsignificant decrease in liver stiffness after 12–24 months. Prednisolone and UDCA, consistent with past studies, were the predominant treatments in our cohort [2]. Although 75%–79% of the patients treated with prednisolone achieved a significant reduction of more than 25% of the baseline cholestasis parameters after 3–12 months, this was achieved in 83%–100% of the patients treated with UDCA. Recently, Graf et al. [2] were also able to demonstrate the potential efficacy of both UDCA and prednisolone in their cohort. In view of the histological detection of bile duct irregularities in our cohort and the possible side effects of immunosuppressive treatment, it seems feasible to apply UDCA as first‐line therapy in patients with mild hepatic sarcoid disease. Additive therapy with prednisolone can be considered in case of lack of response or extrahepatic manifestations.

Data on the progression and development of clinical complications are scarce in patients receiving therapy for hepatic sarcoidosis. Previously, Graf et al. [2] were able to show that the majority of patients undergoing treatment had a stable course. A correlation between lack of laboratory response and progression to portal hypertension could not be confirmed. The data presented here suggest that although therapy for hepatic sarcoidosis leads to a good laboratory response, a small number of patients show a progression of their disease, irrespective of therapy or laboratory response. This demonstrates the great need for new, targeted treatment aims and for outcome studies of patients with hepatic sarcoidosis in prospective multi‐center registries.

Our study has relevant limitations: First, patients with baseline cirrhosis did not undergo TE, preventing us from assessing its diagnostic value for staging hepatic sarcoidosis. Second, treatment regimens were administered at physicians' discretion, mainly pulmonologists, due to prevailing pulmonary disease manifestation, precluding in‐depth assessment of treatment efficacy. Third, for a rather slowly progressive disease such as hepatic sarcoidosis, the observation period was probably too short to detect endpoints such as normalisation of liver enzymes or relevant changes in liver stiffness. Fourth, a selection bias due to loss of follow‐up in the cohort cannot be ruled out, as patients with a clinically favourable course in particular might not attend follow‐up visits.

In conclusion, hepatic sarcoidosis can lead to serious complications such as liver cirrhosis, portal hypertension, or affection of bile ducts. Early introduction of therapy leads to biochemical improvement in most cases. However, its impact on prognosis is still unclear. Initial therapy with UDCA appears to be reasonable in patients with bile duct changes, and a mild disease course and additional therapy with prednisolone may be considered if necessary. Prospective registry studies for hepatic sarcoidosis are required for the identification and validation of prognostic markers and the assessment of treatment regimens and their impact on outcomes.

## Author Contributions

L.J.H. and K.Z. conducted data collection and analysis, and drafted the manuscript. J.L. contributed to data collection. S.W. performed the histological evaluation, provided pathological data, and revised the manuscript. S.L., A.W.L., J.S.z.W. and C.S. offered conceptual input and critically revised the manuscript. M.H.W. participated in data collection, conceptualised the study design, supervised the study, and critically revised the manuscript. M.M. and M.S. conceptualised the study design, supervised the study, and critically revised the manuscript. All authors reviewed the manuscript critically and provided constructive comments on its intellectual content.

## Ethics Statement

Approval was obtained from the Ethics Committee of the Hamburg.

Medical Association (2024‐101318‐BO‐ff) and from the Institutional Review Board of the University Hospital Regensburg (24‐3692‐104).

## Consent

In line with the ethics approval and due to local law (§12 HmbKHG for Hamburg and Art. 27 IV 1 BayKrG for Regensburg), no informed consent is required for retrospective data analysis of patients from Hamburg and patients from Regensburg.

## Conflicts of Interest

The authors declare no conflicts of interest.

## Supporting information


Data S1.

## Data Availability

The data that support the findings of this study are available from the corresponding author, upon reasonable request.
