# Supplementary material for: Insights Into Hepatic Sarcoidosis: Analysis of Histological Patterns, Hepatic Complications and Therapeutic Approaches
Source: Liver Int. 2025 Feb 19;45(3):e70037. doi: 10.1111/liv.70037 (PMC11837985; doi:10.1111/liv.70037)
Supplement: Supplementary file 1 — Data S1. [file LIV-45-0-s001.doc]

**Supplementary material**

**Supplementary patients and methods**

**Clinical details on patients with surgical liver resection**

In one case, surgery was performed for a liver tumour. Histological workup revealed a highly malignant hepatocellular carcinoma (HCC) and hepatic sarcoidosis.

In a second patient with cholestasis, diagnostic cholangioscopy led to suspicion of cholangiocarcinoma (CCA). A hemihepatectomy was performed. Histological workup revealed sarcoidosis with secondary sclerosing cholangitis. CCA was ruled out histologically. The association of sarcoidosis with SSC has been described in a case report before ^45^.

Another patient underwent exploratory laparoscopy due to abdominal lymphadenopathy and splenomegaly. Biopsies of spleen, liver, and kidney were taken during surgery, revealing multi-organ manifestation of sarcoidosis.

**Supplementary Table 1:** Summary of the absolute and relative event rate of histological, clinical and therapeutic characteristics, as well as the corresponding proportion of patients with available data from the total cohort or sub-cohort.

**Supplementary Table 2:** Correlation of granuloma localisation (lobular, portal/periportal, bile duct-associated) with AST, ALT, GGT, ALP, and transient elastography (TE)

**Supplementary Table 3:** Comparison of median levels of AST, ALT, GGT and ALP between baseline and month 3 – 12 follow-up depending on therapy.

**Supplementary Table 4:** Overview of medication at initial hepatological consultation.

**Supplementary Table 5:** **Demographic and clinical characteristics of male and female patients with hepatic sarcoidosis (**AST/ALT: ULN 50 U/L (♂), 35 U/L (♀); GGT: ULN 65 U/L (♂), 38 U/L (♀); ALP: ULN 129 U/L (♂), 104 U/L (♀)).


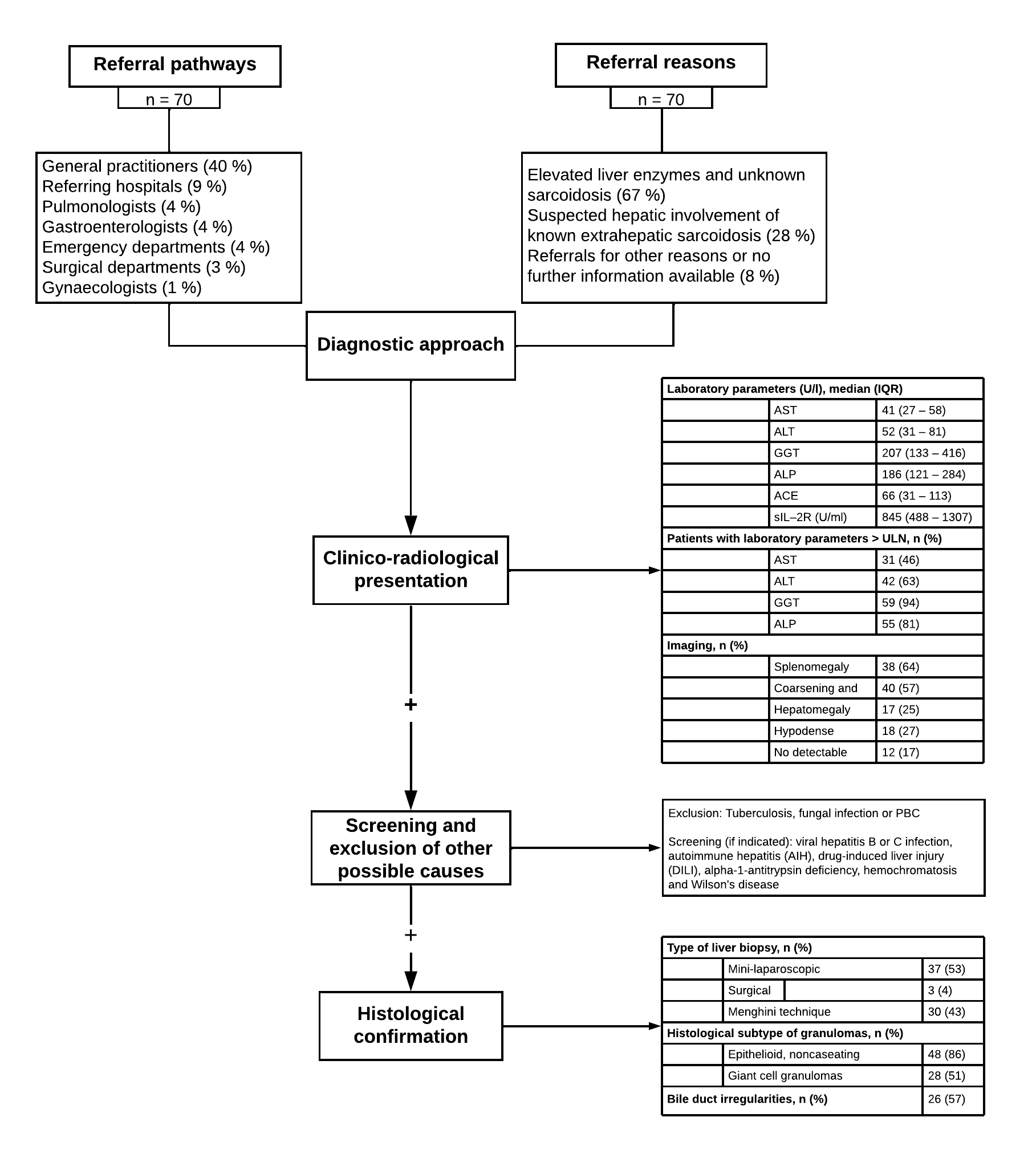
 Supplementary Figure 1: Diagnostic workflow and referral pathways employed for establishing a diagnosis of hepatic sarcoidosis in our patient cohort, incorporating clinical and radiological assessments, exclusion of other liver diseases, and histopathological confirmation.


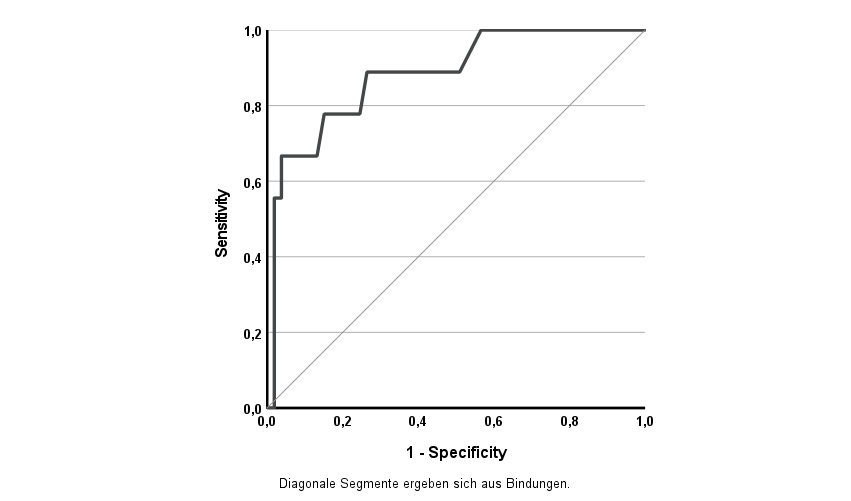


Supplementary Figure 2: ROC curve of FIB-4 score for predicting portal hypertension in patients with hepatic sarcoidosis.
